# Bis{1,4-bis­[(3-butyl­imidazolium-1-yl)meth­yl]benzene}­silver(I) bis­(hexa­fluoridophosphate)

**DOI:** 10.1107/S1600536810036524

**Published:** 2010-09-25

**Authors:** Rosenani A. Haque, Abbas Washeel, Siang Guan Teoh, Madhukar Hemamalini, Hoong-Kun Fun

**Affiliations:** a School of Chemical Sciences, Universiti Sains Malaysia, 11800 USM, Penang, Malaysia; b X-ray Crystallography Unit, School of Physics, Universiti Sains Malaysia, 11800 USM, Penang, Malaysia

## Abstract

The asymmetric unit of the title complex, [Ag_2_(C_22_H_30_N_4_)_2_](PF_6_)_2_, consists of one Ag^I^ ion, one 1,4-bis­[(3-butyl­imidazolium-1-yl)meth­yl]benzene ligand and one discrete hexa­fluoridophosphate anion. The formula unit is generated by an inversion center. The unique Ag^I^ ion is coordinated by two C atoms of two heterocyclic carbene ligands in an essentially linear geometry. In the crystal structure, cations and anions are linked through weak C—H⋯F hydrogen bonds, forming a three-dimensional network.

## Related literature

For applications of *N*-heterocyclic carbenes, see: Tryg *et al.* (2005 ▶); Herrmann (2002 ▶); Herrmann *et al.* (1998 ▶); McGuinness *et al.* (1999 ▶); Tominaga *et al.* (2004 ▶); Magill *et al.* (2001 ▶); Yongbo *et al.* (2008 ▶); Garrison & Youngs (2005 ▶); Kascatan-Nebioglu *et al.* (2007 ▶); Özdemir *et al.* (2010 ▶); Medvetz *et al.* (2008 ▶); Catalano & Malwitz (2003 ▶). For a related structure, see: Chen & Liu (2003 ▶). For the stability of the temperature controller used in the data collection, see: Cosier & Glazer (1986 ▶). 
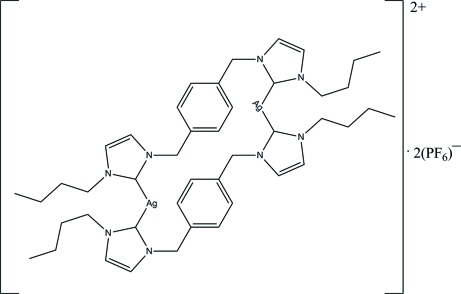


## Experimental

### 

#### Crystal data

[Ag_2_(C_22_H_30_N_4_)_2_](PF_6_)_2_
*M* *_r_* = 1206.68
Triclinic, 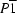

*a* = 11.3636 (15) Å
*b* = 11.4119 (15) Å
*c* = 11.9918 (15) Å
α = 63.528 (2)°
β = 89.335 (2)°
γ = 65.811 (2)°
*V* = 1241.7 (3) Å^3^
*Z* = 1
Mo *K*α radiation
μ = 0.94 mm^−1^
*T* = 100 K
0.24 × 0.14 × 0.08 mm

#### Data collection

Bruker APEXII DUO CCD area-detector diffractometer
Absorption correction: multi-scan (*SADABS*; Bruker, 2009 ▶) *T*_min_ = 0.806, *T*_max_ = 0.930
25433 measured reflections
7142 independent reflections
6512 reflections with *I* > 2σ(*I*)
*R*_int_ = 0.035

#### Refinement

*R*[*F* ^2^ > 2σ(*F* ^2^)] = 0.051
*wR*(*F* ^2^) = 0.161
*S* = 1.16
7142 reflections
309 parameters
H-atom parameters constrained
Δρ_max_ = 3.16 e Å^−3^
Δρ_min_ = −1.23 e Å^−3^

### 

Data collection: *APEX2* (Bruker, 2009 ▶); cell refinement: *SAINT* (Bruker, 2009 ▶); data reduction: *SAINT*; program(s) used to solve structure: *SHELXTL* (Sheldrick, 2008 ▶); program(s) used to refine structure: *SHELXTL*; molecular graphics: *SHELXTL*; software used to prepare material for publication: *SHELXTL* and *PLATON* (Spek, 2009 ▶).

## Supplementary Material

Crystal structure: contains datablocks global, I. DOI: 10.1107/S1600536810036524/lh5130sup1.cif

Structure factors: contains datablocks I. DOI: 10.1107/S1600536810036524/lh5130Isup2.hkl

Additional supplementary materials:  crystallographic information; 3D view; checkCIF report

## Figures and Tables

**Table 1:** Hydrogen-bond geometry (Å, °)

*D*—H⋯*A*	*D*—H	H⋯*A*	*D*⋯*A*	*D*—H⋯*A*
C2—H2*A*⋯F3^i^	0.93	2.42	3.251 (6)	149
C5—H5*A*⋯F1^ii^	0.93	2.52	3.392 (6)	157
C7—H7*B*⋯F5^ii^	0.97	2.44	3.367 (6)	160
C11—H11*A*⋯F6^iii^	0.97	2.44	3.364 (7)	159
C11—H11*B*⋯F2^iv^	0.97	2.38	3.129 (7)	134

Symmetry codes: (i) 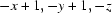; (ii) 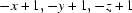; (iii) 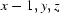; (iv) 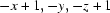.

**Fig. 1.**
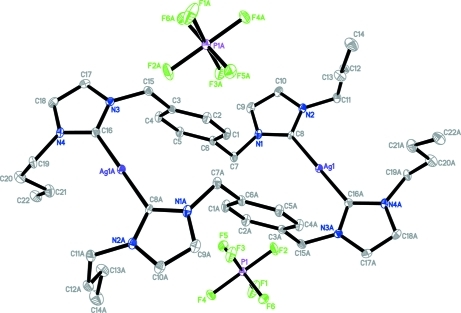
The molecular structure of the title compound, showing 30% probability displacement ellipsoids and the atom-numbering scheme. N1A–N4A/C1A–C22A/P1A and F1A–F6A are generated by the symmetry code -x+1, -y+1, -z (H atoms are omitted for clarity).

**Fig. 2.**
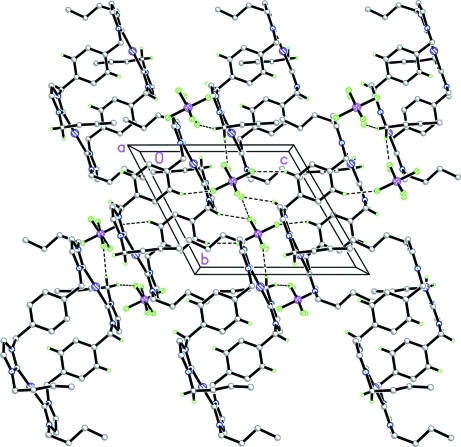
The crystal packing of the title compound, showing hydrogen bonds as dashed lines.

## supplementary crystallographic information

### Comment

*N*-heterocyclic carbenes (NHCs) are now ubiquitous in their usage as ligands for transition metals (Tryg *et al.*, 2005; Herrmann, 2002). Carbene ligands have some similarities to phosphine ligands, but metal- carbene complexes are often more stable than similar metal phosphine complexes (Herrmann *et al.*, 1998; McGuinness *et al.*, 1999). *N*-heterocyclic carbene complexes of different metals such as Pd and Ru have been used as catalysts for many reactions; for example C–C coupling reactions and reactions involving olefin metathesis (Tominaga *et al.*, 2004; Magill *et al.*, 2001).  Among these metal -NHC complexes, the family of silver -NHC complexes have been receiving continuous attention since they are often used as convenient carbene transfer reagents to make other metal-NHC complexes (Yongbo *et al.*, 2008). The chemistry of silver-NHC complexes has been recently reviewed (Garrison & Youngs, 2005; Kascatan-Nebioglu *et al.*, 2007). The biological activity of many of the silver-NHC complexes as antimicrobial and antitumour were also confirmed (Özdemir *et al.*, 2010; Medvetz *et al.*, 2008).

The asymmetric unit of the title compound, (I), consists of one Ag^I^ cation, one 1,4-bis(3-butylimidazolium-1-yl-methyl)benzene ligand and one discrete hexafluoridophosphate anion (Fig. 1). The other half of the title complex is generated by an inversion center (1/2, 1/2, 0). Each Ag^I^ cation is bis-coordinated by two 1,4-bis(3-butylimidazolium-1-yl-methyl)benzene ligands. and displays an essentially linear geometry. The Ag1–C8 = 2.089 (4) Å bond length is comparable to the values reported for other [Ag(carbene)_2_]^+^ complexes (Chen & Liu, 2003).

In the crystal structure, the cations and anions are linked together through intermolecular C2—H2A···F3; C5—H5A···F1; C7—H7B···F5; C11—H11A···F6 and C11—H11B···F2 hydrogen bonds, forming a three-dimensional network (Table 2 and Fig. 2).

### Experimental

Silver oxide, Ag_2_O, (0.13 g, 0.56 mmol) was added to a solution of 1,4-bis(3-butylimidazolium-1-yl-metyl)benzene bis(hexafluoroposphate) (0.30 g, 0.467 mmol) in acetonitrile (40 ml). The mixture was refluxed at (343–363)K for 20 hr in glassware wrapped with aluminium foil to exclude the light. The resulting mixture was filtered through celite to remove excess Ag_2_O. After evaporation of the solvent, the white residue was washed with diethyl ether (2X5 ml) to afford the complex as a white powder. The yield was (0.25g, 45%), m.p = 550–552K. Crystal suitable for X-ray was obtained by slow evaporation of the salt solution in acetonitrile at 281K.

### Refinement

All H atoms were positioned geometrically with C–H = 0.93–0.97 Å and were refined using a riding model, with *U*_iso_(H) = 1.2 or 1.5 *U*_eq_(C). The highest peak in the final difference map is found at a distance of 0.87 Å from C8 and the deepest trough is  1.37 Å from H22C.

### Crystal data

**Table:**

[Ag_2_(C_22_H_30_N_4_)_2_](PF_6_)_2_	*Z* = 1
*M**_r_* = 1206.68	*F*(000) = 612
Triclinic, *P*1	*D*_x_ = 1.614 Mg m^−^^3^
Hall symbol: -P 1	Mo *K*α radiation, λ = 0.71073 Å
*a* = 11.3636 (15) Å	Cell parameters from 9946 reflections
*b* = 11.4119 (15) Å	θ = 2.5–30.1°
*c* = 11.9918 (15) Å	µ = 0.94 mm^−^^1^
α = 63.528 (2)°	*T* = 100 K
β = 89.335 (2)°	Block, colourless
γ = 65.811 (2)°	0.24 × 0.14 × 0.08 mm
*V* = 1241.7 (3) Å^3^	

### Data collection

**Table:**

Bruker APEXII DUO CCD area-detector diffractometer	7142 independent reflections
Radiation source: fine-focus sealed tube	6512 reflections with *I* > 2σ(*I*)
graphite	*R*_int_ = 0.035
φ and ω scans	θ_max_ = 30.0°, θ_min_ = 1.9°
Absorption correction: multi-scan (*SADABS*; Bruker, 2009)	*h* = −15→15
*T*_min_ = 0.806, *T*_max_ = 0.930	*k* = −16→16
25433 measured reflections	*l* = −16→16

### Refinement

**Table:**

Refinement on *F*^2^	Primary atom site location: structure-invariant direct methods
Least-squares matrix: full	Secondary atom site location: difference Fourier map
*R*[*F*^2^ > 2σ(*F*^2^)] = 0.051	Hydrogen site location: inferred from neighbouring sites
*wR*(*F*^2^) = 0.161	H-atom parameters constrained
*S* = 1.16	*w* = 1/[σ^2^(*F*_o_^2^) + (0.0678*P*)^2^ + 6.8955*P*] where *P* = (*F*_o_^2^ + 2*F*_c_^2^)/3
7142 reflections	(Δ/σ)_max_ < 0.001
309 parameters	Δρ_max_ = 3.16 e Å^−^^3^
0 restraints	Δρ_min_ = −1.23 e Å^−^^3^

### Special details

**Table:**

Experimental. The crystal was placed in the cold stream of an Oxford Cryosystems Cobra open-flow nitrogen cryostat (Cosier & Glazer, 1986) operating at 100.0 (1) K.
Geometry. All esds (except the esd in the dihedral angle between two l.s. planes) are estimated using the full covariance matrix. The cell esds are taken into account individually in the estimation of esds in distances, angles and torsion angles; correlations between esds in cell parameters are only used when they are defined by crystal symmetry. An approximate (isotropic) treatment of cell esds is used for estimating esds involving l.s. planes.
Refinement. Refinement of F^2^ against ALL reflections. The weighted R-factor wR and goodness of fit S are based on F^2^, conventional R-factors R are based on F, with F set to zero for negative F^2^. The threshold expression of F^2^ > 2sigma(F^2^) is used only for calculating R-factors(gt) etc. and is not relevant to the choice of reflections for refinement. R-factors based on F^2^ are statistically about twice as large as those based on F, and R- factors based on ALL data will be even larger.

### Fractional atomic coordinates and isotropic or equivalent isotropic displacement parameters (Å^2^)

**Table:**

	*x*	*y*	*z*	*U*_iso_*/*U*_eq_	
Ag1	0.46248 (3)	0.12904 (3)	0.32515 (3)	0.01570 (10)	
N1	0.2537 (4)	0.4313 (4)	0.2939 (3)	0.0193 (7)	
N2	0.1692 (4)	0.2838 (4)	0.3325 (3)	0.0185 (7)	
N3	0.2430 (3)	1.0310 (4)	−0.3191 (3)	0.0170 (6)	
N4	0.3348 (4)	1.1721 (4)	−0.3546 (3)	0.0172 (6)	
C1	0.3370 (5)	0.5534 (5)	0.0323 (4)	0.0238 (9)	
H1A	0.3746	0.4529	0.0615	0.029*	
C2	0.3085 (5)	0.6488 (5)	−0.0971 (4)	0.0237 (9)	
H2A	0.3260	0.6117	−0.1538	0.028*	
C3	0.2540 (4)	0.7998 (5)	−0.1432 (4)	0.0176 (7)	
C4	0.2269 (5)	0.8525 (5)	−0.0564 (4)	0.0221 (8)	
H4A	0.1903	0.9529	−0.0855	0.026*	
C5	0.2544 (5)	0.7555 (5)	0.0743 (4)	0.0214 (8)	
H5A	0.2349	0.7922	0.1311	0.026*	
C6	0.3102 (4)	0.6055 (4)	0.1198 (4)	0.0182 (7)	
C7	0.3438 (5)	0.4986 (5)	0.2612 (4)	0.0212 (8)	
H7A	0.4339	0.4226	0.2846	0.025*	
H7B	0.3375	0.5499	0.3090	0.025*	
C8	0.2850 (4)	0.2935 (4)	0.3183 (3)	0.0138 (6)	
C9	0.1215 (5)	0.5068 (5)	0.2917 (4)	0.0239 (9)	
H9A	0.0778	0.6033	0.2761	0.029*	
C10	0.0679 (5)	0.4132 (5)	0.3166 (4)	0.0241 (9)	
H10A	−0.0192	0.4324	0.3219	0.029*	
C11	0.1508 (5)	0.1543 (5)	0.3571 (4)	0.0230 (8)	
H11A	0.0978	0.1383	0.4212	0.028*	
H11B	0.2362	0.0685	0.3908	0.028*	
C12	0.0843 (5)	0.1712 (5)	0.2381 (4)	0.0247 (9)	
H12A	−0.0006	0.2576	0.2044	0.030*	
H12B	0.0682	0.0869	0.2616	0.030*	
C13	0.1641 (5)	0.1852 (6)	0.1332 (5)	0.0304 (10)	
H13A	0.1737	0.2743	0.1038	0.036*	
H13B	0.2516	0.1027	0.1680	0.036*	
C14	0.0986 (7)	0.1891 (8)	0.0216 (6)	0.0434 (14)	
H14A	0.1528	0.1942	−0.0407	0.065*	
H14B	0.0139	0.2737	−0.0160	0.065*	
H14C	0.0874	0.1019	0.0506	0.065*	
C15	0.2246 (4)	0.9007 (5)	−0.2858 (4)	0.0199 (8)	
H15A	0.2817	0.8464	−0.3245	0.024*	
H15B	0.1342	0.9315	−0.3213	0.024*	
C16	0.3620 (4)	1.0335 (5)	−0.3299 (4)	0.0186 (7)	
C17	0.1448 (4)	1.1638 (5)	−0.3387 (4)	0.0229 (8)	
H17A	0.0565	1.1870	−0.3363	0.027*	
C18	0.2016 (4)	1.2543 (5)	−0.3621 (4)	0.0227 (8)	
H18A	0.1600	1.3516	−0.3796	0.027*	
C19	0.4337 (5)	1.2264 (5)	−0.3649 (4)	0.0220 (8)	
H19A	0.4153	1.3055	−0.4508	0.026*	
H19B	0.5203	1.1479	−0.3498	0.026*	
C20	0.4346 (6)	1.2817 (6)	−0.2701 (5)	0.0320 (10)	
H20A	0.5015	1.3158	−0.2814	0.038*	
H20B	0.3500	1.3657	−0.2911	0.038*	
C21	0.4601 (6)	1.1718 (6)	−0.1314 (5)	0.0320 (10)	
H21A	0.5518	1.0988	−0.1039	0.038*	
H21B	0.4060	1.1219	−0.1217	0.038*	
C22	0.4300 (6)	1.2429 (6)	−0.0469 (5)	0.0317 (10)	
H22A	0.4439	1.1698	0.0396	0.048*	
H22B	0.3398	1.3169	−0.0748	0.048*	
H22C	0.4872	1.2871	−0.0519	0.048*	
P1	0.76781 (11)	0.28501 (11)	0.51153 (10)	0.0165 (2)	
F1	0.8121 (5)	0.2072 (4)	0.6636 (3)	0.0521 (11)	
F2	0.6958 (3)	0.1871 (4)	0.5238 (4)	0.0452 (9)	
F3	0.7303 (4)	0.3610 (3)	0.3605 (3)	0.0399 (8)	
F4	0.8417 (3)	0.3835 (3)	0.4983 (3)	0.0347 (7)	
F5	0.6356 (3)	0.4085 (3)	0.5124 (3)	0.0326 (7)	
F6	0.9021 (3)	0.1590 (3)	0.5128 (3)	0.0294 (6)	

### Atomic displacement parameters (Å^2^)

**Table:**

	*U*^11^	*U*^22^	*U*^33^	*U*^12^	*U*^13^	*U*^23^
Ag1	0.01635 (15)	0.01335 (14)	0.01699 (14)	−0.00615 (11)	0.00410 (10)	−0.00754 (11)
N1	0.0274 (18)	0.0148 (15)	0.0154 (14)	−0.0088 (14)	0.0061 (13)	−0.0076 (12)
N2	0.0216 (17)	0.0157 (15)	0.0168 (15)	−0.0091 (13)	0.0085 (13)	−0.0063 (12)
N3	0.0173 (16)	0.0161 (15)	0.0147 (14)	−0.0069 (13)	0.0025 (12)	−0.0058 (12)
N4	0.0215 (17)	0.0127 (14)	0.0159 (14)	−0.0080 (13)	0.0058 (12)	−0.0056 (12)
C1	0.036 (2)	0.0166 (18)	0.0178 (18)	−0.0101 (17)	0.0053 (16)	−0.0090 (15)
C2	0.035 (2)	0.0196 (19)	0.0167 (17)	−0.0111 (18)	0.0043 (16)	−0.0101 (16)
C3	0.0198 (18)	0.0199 (18)	0.0152 (16)	−0.0115 (15)	0.0034 (14)	−0.0080 (14)
C4	0.030 (2)	0.0160 (18)	0.0188 (18)	−0.0100 (16)	0.0060 (16)	−0.0081 (15)
C5	0.031 (2)	0.0185 (18)	0.0179 (17)	−0.0114 (17)	0.0064 (16)	−0.0110 (15)
C6	0.024 (2)	0.0165 (17)	0.0159 (16)	−0.0113 (15)	0.0051 (14)	−0.0074 (14)
C7	0.032 (2)	0.0172 (18)	0.0157 (17)	−0.0124 (17)	0.0034 (15)	−0.0077 (15)
C8	0.0168 (17)	0.0095 (15)	0.0122 (15)	−0.0040 (13)	0.0044 (12)	−0.0047 (12)
C9	0.029 (2)	0.0188 (19)	0.0211 (19)	−0.0084 (17)	0.0115 (16)	−0.0096 (16)
C10	0.024 (2)	0.0195 (19)	0.024 (2)	−0.0060 (16)	0.0102 (16)	−0.0106 (16)
C11	0.028 (2)	0.0199 (19)	0.0218 (19)	−0.0134 (17)	0.0084 (16)	−0.0078 (16)
C12	0.026 (2)	0.027 (2)	0.024 (2)	−0.0164 (18)	0.0064 (17)	−0.0110 (17)
C13	0.035 (3)	0.041 (3)	0.026 (2)	−0.023 (2)	0.0115 (19)	−0.019 (2)
C14	0.058 (4)	0.055 (4)	0.034 (3)	−0.034 (3)	0.010 (3)	−0.026 (3)
C15	0.024 (2)	0.0234 (19)	0.0158 (16)	−0.0157 (17)	0.0046 (14)	−0.0080 (15)
C16	0.0218 (19)	0.0181 (18)	0.0155 (16)	−0.0095 (15)	0.0031 (14)	−0.0072 (14)
C17	0.0188 (19)	0.023 (2)	0.0209 (18)	−0.0061 (16)	0.0003 (15)	−0.0092 (16)
C18	0.022 (2)	0.0176 (18)	0.0221 (19)	−0.0029 (16)	0.0036 (15)	−0.0094 (16)
C19	0.032 (2)	0.0203 (19)	0.0213 (18)	−0.0178 (18)	0.0104 (16)	−0.0103 (16)
C20	0.039 (3)	0.039 (3)	0.026 (2)	−0.024 (2)	0.009 (2)	−0.016 (2)
C21	0.043 (3)	0.033 (3)	0.028 (2)	−0.022 (2)	0.010 (2)	−0.017 (2)
C22	0.037 (3)	0.042 (3)	0.031 (2)	−0.023 (2)	0.012 (2)	−0.024 (2)
P1	0.0183 (5)	0.0159 (4)	0.0173 (4)	−0.0078 (4)	0.0065 (4)	−0.0096 (4)
F1	0.085 (3)	0.0284 (16)	0.0167 (13)	−0.0055 (18)	0.0095 (16)	−0.0087 (12)
F2	0.0351 (18)	0.0304 (16)	0.087 (3)	−0.0241 (15)	0.0275 (18)	−0.0333 (19)
F3	0.068 (2)	0.0251 (15)	0.0168 (12)	−0.0126 (15)	0.0001 (13)	−0.0101 (11)
F4	0.0286 (15)	0.0293 (15)	0.057 (2)	−0.0171 (13)	0.0074 (14)	−0.0260 (15)
F5	0.0254 (15)	0.0274 (14)	0.0473 (18)	−0.0080 (12)	0.0142 (13)	−0.0235 (14)
F6	0.0217 (13)	0.0262 (14)	0.0408 (16)	−0.0056 (11)	0.0103 (12)	−0.0214 (13)

### Geometric parameters (Å, °)

**Table:**

Ag1—C16^i^	2.073 (4)	C12—C13	1.531 (7)
Ag1—C8	2.089 (4)	C12—H12A	0.9700
N1—C8	1.348 (5)	C12—H12B	0.9700
N1—C9	1.386 (6)	C13—C14	1.514 (7)
N1—C7	1.470 (6)	C13—H13A	0.9700
N2—C8	1.367 (5)	C13—H13B	0.9700
N2—C10	1.379 (5)	C14—H14A	0.9600
N2—C11	1.476 (6)	C14—H14B	0.9600
N3—C16	1.368 (5)	C14—H14C	0.9600
N3—C17	1.379 (6)	C15—H15A	0.9700
N3—C15	1.463 (5)	C15—H15B	0.9700
N4—C16	1.367 (5)	C16—Ag1^i^	2.073 (4)
N4—C18	1.394 (6)	C17—C18	1.360 (7)
N4—C19	1.470 (6)	C17—H17A	0.9300
C1—C2	1.384 (6)	C18—H18A	0.9300
C1—C6	1.395 (6)	C19—C20	1.530 (7)
C1—H1A	0.9300	C19—H19A	0.9700
C2—C3	1.394 (6)	C19—H19B	0.9700
C2—H2A	0.9300	C20—C21	1.509 (7)
C3—C4	1.392 (6)	C20—H20A	0.9700
C3—C15	1.518 (5)	C20—H20B	0.9700
C4—C5	1.400 (6)	C21—C22	1.516 (7)
C4—H4A	0.9300	C21—H21A	0.9700
C5—C6	1.384 (6)	C21—H21B	0.9700
C5—H5A	0.9300	C22—H22A	0.9600
C6—C7	1.516 (6)	C22—H22B	0.9600
C7—H7A	0.9700	C22—H22C	0.9600
C7—H7B	0.9700	P1—F5	1.588 (3)
C9—C10	1.359 (7)	P1—F3	1.590 (3)
C9—H9A	0.9300	P1—F2	1.594 (3)
C10—H10A	0.9300	P1—F1	1.606 (3)
C11—C12	1.518 (6)	P1—F6	1.606 (3)
C11—H11A	0.9700	P1—F4	1.613 (3)
C11—H11B	0.9700		
			
C16^i^—Ag1—C8	179.40 (15)	C12—C13—H13B	109.3
C8—N1—C9	111.3 (4)	H13A—C13—H13B	107.9
C8—N1—C7	124.8 (4)	C13—C14—H14A	109.5
C9—N1—C7	123.7 (4)	C13—C14—H14B	109.5
C8—N2—C10	111.7 (4)	H14A—C14—H14B	109.5
C8—N2—C11	125.2 (4)	C13—C14—H14C	109.5
C10—N2—C11	123.1 (4)	H14A—C14—H14C	109.5
C16—N3—C17	111.9 (4)	H14B—C14—H14C	109.5
C16—N3—C15	123.6 (4)	N3—C15—C3	113.3 (3)
C17—N3—C15	124.5 (4)	N3—C15—H15A	108.9
C16—N4—C18	111.5 (4)	C3—C15—H15A	108.9
C16—N4—C19	124.8 (4)	N3—C15—H15B	108.9
C18—N4—C19	123.6 (4)	C3—C15—H15B	108.9
C2—C1—C6	121.1 (4)	H15A—C15—H15B	107.7
C2—C1—H1A	119.4	N4—C16—N3	103.5 (4)
C6—C1—H1A	119.4	N4—C16—Ag1^i^	128.5 (3)
C1—C2—C3	120.6 (4)	N3—C16—Ag1^i^	127.8 (3)
C1—C2—H2A	119.7	C18—C17—N3	106.8 (4)
C3—C2—H2A	119.7	C18—C17—H17A	126.6
C4—C3—C2	118.5 (4)	N3—C17—H17A	126.6
C4—C3—C15	122.5 (4)	C17—C18—N4	106.2 (4)
C2—C3—C15	119.0 (4)	C17—C18—H18A	126.9
C3—C4—C5	120.6 (4)	N4—C18—H18A	126.9
C3—C4—H4A	119.7	N4—C19—C20	112.2 (4)
C5—C4—H4A	119.7	N4—C19—H19A	109.2
C6—C5—C4	120.7 (4)	C20—C19—H19A	109.2
C6—C5—H5A	119.6	N4—C19—H19B	109.2
C4—C5—H5A	119.6	C20—C19—H19B	109.2
C5—C6—C1	118.4 (4)	H19A—C19—H19B	107.9
C5—C6—C7	121.5 (4)	C21—C20—C19	115.9 (4)
C1—C6—C7	120.1 (4)	C21—C20—H20A	108.3
N1—C7—C6	110.8 (3)	C19—C20—H20A	108.3
N1—C7—H7A	109.5	C21—C20—H20B	108.3
C6—C7—H7A	109.5	C19—C20—H20B	108.3
N1—C7—H7B	109.5	H20A—C20—H20B	107.4
C6—C7—H7B	109.5	C20—C21—C22	112.4 (5)
H7A—C7—H7B	108.1	C20—C21—H21A	109.1
N1—C8—N2	104.1 (3)	C22—C21—H21A	109.1
N1—C8—Ag1	130.4 (3)	C20—C21—H21B	109.1
N2—C8—Ag1	125.4 (3)	C22—C21—H21B	109.1
C10—C9—N1	107.0 (4)	H21A—C21—H21B	107.9
C10—C9—H9A	126.5	C21—C22—H22A	109.5
N1—C9—H9A	126.5	C21—C22—H22B	109.5
C9—C10—N2	105.9 (4)	H22A—C22—H22B	109.5
C9—C10—H10A	127.1	C21—C22—H22C	109.5
N2—C10—H10A	127.1	H22A—C22—H22C	109.5
N2—C11—C12	112.6 (4)	H22B—C22—H22C	109.5
N2—C11—H11A	109.1	F5—P1—F3	90.87 (19)
C12—C11—H11A	109.1	F5—P1—F2	91.02 (18)
N2—C11—H11B	109.1	F3—P1—F2	90.1 (2)
C12—C11—H11B	109.1	F5—P1—F1	90.93 (19)
H11A—C11—H11B	107.8	F3—P1—F1	177.6 (2)
C11—C12—C13	114.1 (4)	F2—P1—F1	91.5 (2)
C11—C12—H12A	108.7	F5—P1—F6	179.20 (18)
C13—C12—H12A	108.7	F3—P1—F6	89.93 (18)
C11—C12—H12B	108.7	F2—P1—F6	89.00 (17)
C13—C12—H12B	108.7	F1—P1—F6	88.27 (19)
H12A—C12—H12B	107.6	F5—P1—F4	89.49 (17)
C14—C13—C12	111.8 (5)	F3—P1—F4	89.6 (2)
C14—C13—H13A	109.3	F2—P1—F4	179.4 (2)
C12—C13—H13A	109.3	F1—P1—F4	88.8 (2)
C14—C13—H13B	109.3	F6—P1—F4	90.49 (17)
			
C6—C1—C2—C3	1.0 (8)	C11—N2—C10—C9	−177.9 (4)
C1—C2—C3—C4	−1.0 (7)	C8—N2—C11—C12	−101.3 (5)
C1—C2—C3—C15	179.8 (4)	C10—N2—C11—C12	76.3 (5)
C2—C3—C4—C5	0.2 (7)	N2—C11—C12—C13	63.3 (5)
C15—C3—C4—C5	179.3 (4)	C11—C12—C13—C14	175.3 (5)
C3—C4—C5—C6	0.7 (7)	C16—N3—C15—C3	85.2 (5)
C4—C5—C6—C1	−0.8 (7)	C17—N3—C15—C3	−92.3 (5)
C4—C5—C6—C7	178.5 (4)	C4—C3—C15—N3	34.6 (6)
C2—C1—C6—C5	−0.1 (7)	C2—C3—C15—N3	−146.2 (4)
C2—C1—C6—C7	−179.3 (5)	C18—N4—C16—N3	−1.5 (4)
C8—N1—C7—C6	103.8 (4)	C19—N4—C16—N3	175.3 (3)
C9—N1—C7—C6	−69.9 (5)	C18—N4—C16—Ag1^i^	173.8 (3)
C5—C6—C7—N1	109.1 (5)	C19—N4—C16—Ag1^i^	−9.4 (6)
C1—C6—C7—N1	−71.7 (5)	C17—N3—C16—N4	1.1 (4)
C9—N1—C8—N2	−0.4 (4)	C15—N3—C16—N4	−176.7 (3)
C7—N1—C8—N2	−174.9 (3)	C17—N3—C16—Ag1^i^	−174.3 (3)
C9—N1—C8—Ag1	176.5 (3)	C15—N3—C16—Ag1^i^	8.0 (6)
C7—N1—C8—Ag1	2.0 (6)	C16—N3—C17—C18	−0.3 (5)
C10—N2—C8—N1	0.2 (4)	C15—N3—C17—C18	177.4 (4)
C11—N2—C8—N1	178.1 (4)	N3—C17—C18—N4	−0.6 (5)
C10—N2—C8—Ag1	−176.8 (3)	C16—N4—C18—C17	1.4 (5)
C11—N2—C8—Ag1	1.0 (5)	C19—N4—C18—C17	−175.4 (4)
C8—N1—C9—C10	0.5 (5)	C16—N4—C19—C20	−122.3 (5)
C7—N1—C9—C10	175.0 (4)	C18—N4—C19—C20	54.0 (6)
N1—C9—C10—N2	−0.3 (5)	N4—C19—C20—C21	58.0 (6)
C8—N2—C10—C9	0.0 (5)	C19—C20—C21—C22	−167.5 (5)

Symmetry codes: (i) −*x*+1, −*y*+1, −*z*.

### Hydrogen-bond geometry (Å, °)

**Table:**

*D*—H···*A*	*D*—H	H···*A*	*D*···*A*	*D*—H···*A*
C2—H2A···F3^i^	0.93	2.42	3.251 (6)	149
C5—H5A···F1^ii^	0.93	2.52	3.392 (6)	157
C7—H7B···F5^ii^	0.97	2.44	3.367 (6)	160
C11—H11A···F6^iii^	0.97	2.44	3.364 (7)	159
C11—H11B···F2^iv^	0.97	2.38	3.129 (7)	134

Symmetry codes: (i) −*x*+1, −*y*+1, −*z*; (ii) −*x*+1, −*y*+1, −*z*+1; (iii) *x*−1, *y*, *z*; (iv) −*x*+1, −*y*, −*z*+1.
